# Association of Accelerometer-Measured Sedentary Time and Physical Activity with Arterial Stiffness and Vascular Aging in the General Spanish Population, Analyzed by Sex

**DOI:** 10.31083/j.rcm2411318

**Published:** 2023-11-17

**Authors:** Leticia Gómez-Sánchez, Marta Gómez-Sánchez, Emiliano Rodríguez-Sánchez, Cristina Lugones-Sánchez, Olaya Tamayo-Morales, Susana Gonzalez-Sánchez, Angela de Cabo-Laso, Luis García-Ortiz, Manuel A. Gómez-Marcos, on behalf of the EVA Investigators

**Affiliations:** ^1^Primary Care Research Unit of Salamanca (APISAL), Health Service of Castile and Leon (SACyL), 37005 Salamanca, Spain; ^2^Department of Medicine, University of Salamanca, 37007 Salamanca, Spain; ^3^Biomedical Research Institute of Salamanca (IBSAL), 37007 Salamanca, Spain; ^4^Research Network in Chronicity, Primary Care and Health Promotion (RICAPPS) (RD21/0016), 28041 Barcelona, Spain; ^5^Department of Nurse, University of Salamanca, 37007 Salamanca, Spain; ^6^Department of Biomedical and Diagnostic Sciences, University of Salamanca, 37007 Salamanca, Spain

**Keywords:** sedentary time, physical activity, accelerometer, arterial stiffness, vascular aging, Spanish population

## Abstract

**Background:**

In this study we analyzed the association between physical activity and sedentary lifestyle with vascular aging in Spanish populations aged 35–75 years.

**Methods:**

A cross-sectional study was developed, in which 501 subjects aged 35–75 years were recruited. Physical activity and sedentary time were measured with an accelerometer (Actigraph GTX3) for a week. We measured carotid–femoral pulse wave velocity (cfPWV) by a Sphygmo Cor® device and carotid intima-media thickness (cIMT) by ultrasound (Sonosite Micromax®). The vascular aging index (VAI) was calculated as described in the literature. Vascular aging was defined considering the 25th and 75th percentiles by age and sex of cfPWV and VAI, presence of vascular injury, type-2 diabetes mellitus or arterial hypertension. Individuals were classified into three groups: healthy, normal, and early vascular aging.

**Results:**

The mean age of the sample was 55.90 ± 14.24 years, 50% being women. Total physical activity was negatively associated with cfPWV (β = –0.454) and VAI (β = –1.845). Similarly, the number of steps per day obtained a negative association with cfPWV (β = –0.052) and VAI (β = –0.216), while sedentary time showed a positive association with cfPWV (β = 0.028) and VAI (β = 0.117). In the analysis by sex, the results showed similar values. The odds ratio (OR) of total physical activity of subjects classified as early vascular aging (EVA) with regarding those classified as healthy vascular aging (HVA) was 0.521 (95% confidence interval [CI] 0.317 to 0.856) for cfPWV, and 0.565 (95% CI 0.324 to 0.986) for VAI. In terms of the number of steps per day, the OR was 0.931 (95% CI 0.875 to 0.992) for cfPWV and 0.916 (95% CI 0.847 to 0.990) for VAI and for sedentary time the OR was 1.042 (95% CI 1.011 to 1.073) for cfPWV and 1.037 (95% CI 1.003 to 1.072) for VAI. The OR of subjects classified as vigorous physical activity was 0.196 (95% CI 0.041 to 0.941) using cfPWV and 0.161 (95% CI 0.032 to 0.820) using VAI. In the analysis by sex, the results showed an association in men when cfPWV was used and an association in women when VAI was used to define vascular aging.

**Conclusions:**

The results of this study indicate that the more time spent performing physical activity and the less sedentary time, the lower the arterial stiffness and the probability of developing early vascular aging.

**Clinical Trial Registration:**

The study was registered in ClinicalTrials.gov (number: NCT02623894).

## 1. Introduction

Being sedentary and physical inactivity are highly prevalent, affecting one third of the world population [1]. Sedentary behaviours have an adverse impact on health, regardless of physical activity, increasing mortality by all causes, including by cardiovascular diseases, the risk of cancer, the risk of metabolic disorders such as diabetes mellitus, hypertension and dyslipidaemia, the risk of musculoskeletal disorders such as arthralgia and osteoporosis, and the risk of depression and cognitive deterioration [2, 3, 4]. On the contrary, it has been shown that the more time spent doing physical activity, regardless of the type and intensity, the lower the morbidity and mortality due to cardiometabolic diseases, tumours and mental disorders, with an improvement in the quality of life and the benefits for the health system and society [5, 6, 7, 8, 9, 10, 11].

Arterial stiffness, related to age and other risk factors such as hypertension, dyslipidemia and diabetes, which favour the development of atherosclerosis, predicts increased cardiovascular morbidity and mortality [12]. Numerous studies suggest that arterial stiffness is lower in active than in sedentary subjects [13, 14, 15, 16, 17]. The effect of physical activity on arterial stiffness, depending on the intensity and type of physical activity, remains controversial. Several studies suggest that a benefit is only achieved with moderate or vigorous intense activity and can vary according to the type of exercise [18, 19, 20, 21]. Similarly, other studies indicate that the effect varies by sex [21, 22].

Vascular aging depends on the structure and stiffness of the arteries and indicates the discrepancy between the age of the arteries and the chronological age of the individual [23, 24]. The factors influencing vascular aging have been studied recently in numerous studies, and their analysis has found a greater relationship with morbidity and mortality from cardiovascular disease than biological aging [23, 24]. However, there is still no agreed definition of vascular aging and recent work indicates the importance that three-dimensional (3D) imaging can have, as well as vascular permeability [25, 26]. Thus, several authors have defined vascular aging using the percentiles of arterial stiffness measured by carotid–femoral pulse wave velocity (cfPWV) [24, 27, 28, 29, 30, 31], and Nilsson Wadström *et al*. [32] have published an index to evaluate vascular aging, the vascular aging index (VAI). This parameter integrates carotid intima-media thickness (cIMT) and cfPWV, which reflect subclinical arteriosclerosis and arterial stiffness.

Studies have analysed the effect of physical activity and sedentary lifestyles on vascular aging. They have found that physical activity increases nitric oxide (NO) bioavailability and reduces oxidative stress and inflammation in blood vessel walls, which may delay arterial aging [33, 34, 35, 36, 37, 38]. We hypothesise that that subjects who perform more physical activity at higher intensity and are less sedentary will have better vascular health and be less likely to be classified as having early vascular aging.

Given the above, the aim of the present study was to analyse the association of sedentary time and physical activity, as measured objectively using an accelerometer, with arterial stiffness, while also exploring the differences by sex in a Spanish population sample without cardiovascular disease.

## 2. Materials and Methods

### 2.1 Study Design and Population

The association between different risk factors and early vascular aging (EVA study) [39], is cross-sectional study developed in Primary Care in Salamanca (Spain). The study protocol was registered in ClinicalTrials.gov (NCT02623894). The participants were recruited between June 2016 and November 2017 by random sampling, with replacement by age decades (35, 45, 55, 65 and 75 years). A total of 501 individuals were included from a reference population of 43,946 users registered in five urban healthcare centers (100 individuals per age group; 50% of each sex). The participant flowchart with the response rate is shown in **Supplementary Fig. 1**. The inclusion criteria were: age between 35 and 75 years and having signed the informed consent. Exclusion criteria: terminally ill subjects, inability to travel to health centres, history of cardiovascular disease, glomerular filtration rate <30 mL/min/1.73 $m^{2}$, chronic inflammatory disease or an acute inflammatory process in the last 3 months or being treated with estrogen, testosterone or growth hormone [39, 40]. The estimation of the sample size has been done for the main objective of the study. For the sedentary time, with an alpha risk of 0.05 and a beta risk of 0.20 in a two-sided test and a standard deviation (SD) of 567 minutes/week, 140 subjects were necessary in each group to recognize a minimum difference of 220 minutes/week in sedentary time between any pair of three groups exist (healthy vascular aging [HVA], normal vascular aging [NVA] and EVA) as statistically significant. The estimated power of the study was 80%. For the number of steps/day, accepting also an alpha risk of 0.05 and a beta risk of 0.20 in a two-sided test and a SD of 4080 steps/day, 152 subjects were necessary in each group to recognize a minimum difference of 1500 steps/day between any pair of three groups as statistically significant. The estimated power of the study was 73%.

### 2.2 Variables and Measurements

Two previously trained investigators gathered the data, following a standardized protocol. The measurements taken from each participant were carried out within 10 days.

#### 2.2.1 Physical Activity Assessment and Sitting Time

Physical activity was evaluated using the ActiGraph-GT3X accelerometer (ActiGraph, Shalimar, FL, USA) [41]. It is a 3D accelerometer that records and measures linear accelerations in three axes: X, Y, and Z, and was used for seven consecutive days, including the step count and the intensity of the physical activity carried out. The original data from the accelerometers was collected at a frequency of 30 Hz. Accelerometers were attached to the waist, placed on the axillary line at the level of the iliac crest of the right or left hip and worn for seven consecutive days, except during bathing or swimming. The accelerometers recorded activity at 1-min intervals, during the day and night. If the number of days was <3 days per week or the time of use was <8 h per day, the data were classed as invalid. Physical activity (PA) was measured in minutes/week and intensity in basal metabolic rate/minute/week (METs min/week). Participants were classified according to the intensity of physical activity in the categories defined in the World Health Organization 2020 guidelines [42]. Light physical activity is considered to be those who do not reach 150 min/week of moderate-intensity or 75 min/week of vigorous-intensity (<1 MET × h/day); moderate physical activity, those who met recommendations of 150–300 min/week of moderate- or 75–150 min/week of vigorous-intensity (1–3.9 MET × h/day) and vigorous physical activity, those who exceed 300 min/week of moderate- or 150 min/week of vigorous-intensity (≥4 MET × h/day). Light physical activity was considered as the reference category.

#### 2.2.2 Carotid Intima-Media Thickness 

The reliability was assessed prior to the study by intra-class coefficient, showing values of 0.974 for intra-observer agreement in repeated measures in 20 individuals and 0.897 for inter-observer agreement. The measurements were recorded with a Sonosite Micromax® ultrasound (Sonosite Inc., Bothell, Washington, DC, USA), using a high-resolution linear transductor at a multi-frequency of 5–10 MHz. The measurements were taken from the common carotid at 1 cm from the bifurcation. The cIMT was measured at the anterior wall and at the posterior wall and in all cases 3 projections were used: anterior, posterior, and lateral, discriminating two lines, intimate-blood interface and media-adventitia interface. A total of six measurements were obtained from the right carotid and another six from the left carotid, using average values (average cIMT) automatically calculated by the Sonocal software 5.0 (Bothell, Washington, DC, USA) [43].

#### 2.2.3 Arterial Stiffness Measurement, Carotid-Femoral Pulse Wave Velocity

The cfPWV waves were analyzed with the patient in a supine position using the SphygmoCor System (AtCor Medical Pty Ltd., Head Office, West Ryde, Australia). To detect the pulse waves, a sensor was placed in the neck (carotid artery) and another in the groin (femoral artery). Once it records the pulse wave with proper morphology for 11 seconds, the system uses algorithms to calculate the speed at which the wave propagates along the arteries. The cfPWV is estimated considering the lag time with respect to the R wave of the electrocardiogram. To this end, the distance between the sternal notch and the point where the sensor is placed over the carotid and femoral arteries was determined using a measuring tape [44]. The quality of the pulse wave is set by the device-specific software, and the manufacturer’s instructions for estimating cfPWV were always followed. The device provides results of the average pulse wave velocity.

#### 2.2.4 Vascular Aging Index 

The VAI was calculated by using the following formula [32]:

$$\begin{array}{c} \mathrm{VAI}=\left(\mathrm{LN}\,\left(1.09\right)\times 10\,c\,I\,M\,T+\mathrm{LN}\,\left(1.14\right)\times \mathrm{aPWV}\right)\times 39.1+4.76 \end{array}$$

where cIMT is the carotid intima-media thickness, aPWV is the aortic pulse wave velocity equivalent to cfPWV, and LN is the natural logarithm with base e. The VAI is a parameter that combines methods to measure different arterial properties. It takes into account the vascular structure assessed with cIMT, which reflects already established atherosclerosis, and the aPWV equivalent to cfPWV that reflects arterial stiffness [45].

#### 2.2.5 Criteria for Healthy Vascular Aging, Normal Vascular Aging, and Early Vascular Aging 

**Fig. 1.**
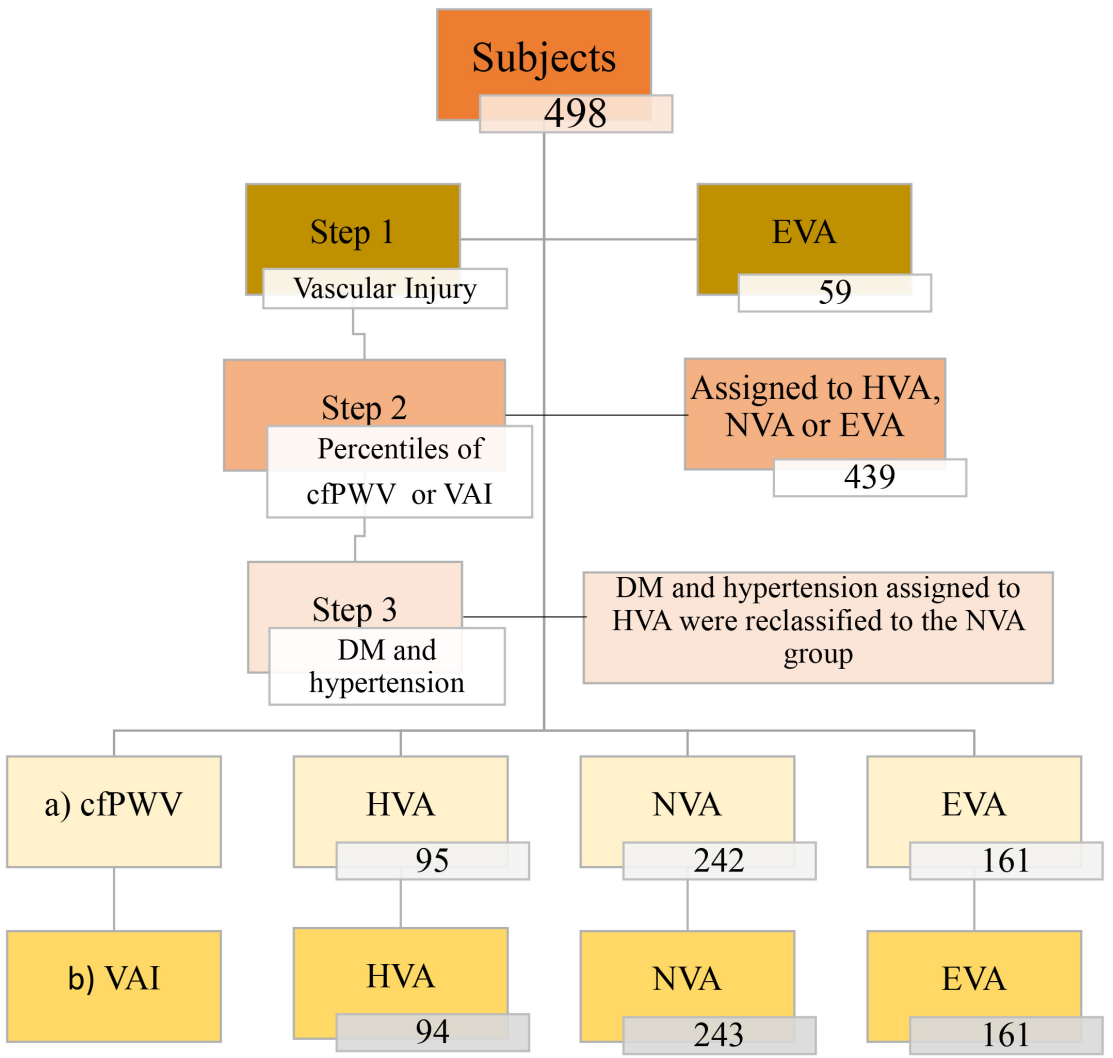
**Distribution of the participants with the two criteria in each of the groups: healthy vascular aging, normal vascular aging, and early vascular aging**. (a) The 25th and 75th cfPWV percentiles of the population studied by age and sex. (b) The 25th and 75th VAI percentiles of the population studied by age and sex. The participants were classified as follows: above the 75th was considered early vascular aging, between the 25th and 75th was considered normal vascular aging, and below the 25th was considered healthy vascular aging. cfPWV, carotid-femoral pulse wave velocity; VAI, vascular aging index; EVA, early vascular aging; HVA, healthy vascular aging; NVA, normal vascular aging; DM, diabetes mellitus type 2.

Vascular aging was defined using two criteria, cfPWV and VAI, following these three steps: (1) 59 participants who presented vascular injury in carotid arteries or peripheral arterial disease were classified as EVA [45]; (2) using the 25th and 75th percentiles of cfPWV and VAI of the analyzed population by age and sex, the individuals were classified into three groups (EVA, values above P75; NVA, values between P25 and P75; HVA, values below P25); (3) participants with hypertension or type-2 diabetes mellitus classified in HVA group were reclassified as NVA. Fig. 1 shows the distribution of the 498 participants, who presented cfPWV and cIMT, according to the degree of vascular aging with the two criteria used in the definition. In **Supplementary Fig. 2** are shown the 25th and 75th percentiles by age and sex of cfPWV (a) and VAI (b) of the subjects of the study.

#### 2.2.6 Ankle-Brachial Index 

Ankle-brachial index (ABI) was measure using a Vasera device VS-1500 (Fukuda Denshi Co., Ltd. Tokyo, Japan). The presence of vascular injury was established following the criteria of the European Society of Hypertension and the European Society of Cardiology [45].

#### 2.2.7 Evaluation of Lifestyles 

Smoking was assessed by a standardized questionnaire (current smoker or not, number of cigarettes consumed, and years of smoking). The participants were defined as smokers if they smoked at the time of evaluation or had stopped smoking within the last year. Alcohol consumption was also assessed by a standardized questionnaire estimated alcohol intake in g/week. Consumption of less than 140 g/week for women and less than 210 g/week for men was considered low risk. Adherence to the Mediterranean diet was evaluated with a 14-item questionnaire, validated in Spain and used in the PREDIMED study [46]. Adherence to the Mediterranean diet was considered for scores equal to or above 9 points. The adherence to the Mediterranean diet questionnaire is shown in **Supplementary Table 1**.

#### 2.2.8 Cardiovascular Risk Factors Definition

We have considered that the subject has hypertension if: systolic blood pressure (BP) ≥140 mmHg, diastolic BP ≥90 mmHg, or use of antihypertensive drugs. Dyslipidemia was defined if: total cholesterol ≥240 mg/dL, low-density lipoprotein (LDL) cholesterol ≥160 mg/dL, high-density lipoprotein (HDL) cholesterol ≤40 mg/dL in men or ≤50 mg/dL in women, triglycerides ≥150 mg/dL, or use of lipid-lowering drugs. Type-2 diabetes mellitus was defined as having one or more of the following conditions: fasting plasma glucose ≥126 mg/dL, glycosylated haemoglobin (HbA1c) ≥6.5%, or use of antidiabetic drugs. Obesity was defined as a body mass index (BMI) >30 kg/$m^{2}$ [45, 47, 48].

#### 2.2.9 Analytical Tests

We measured fasting plasma glucose, total cholesterol, high-density lipoprotein cholesterol and triglyceride levels using a standard enzyme assay. Glycosylated hemoglobin was measured with an automated immuno-turbidimetric assay method. All analytical tests were processed in the same laboratory. The blood sample was obtained in the morning (8–9 h) after a 12-hour fast [39].

### 2.3 Statistical Analysis

The continuous variables are shown as the mean ± SD, and the categorical variables as numbers and percentages. We performed a Student’s *t*-test to compare the means between two independent groups and analyses of variance (ANOVA) when comparing more than two groups. The χ^2^ test was used to compare the categorical variables. To explore the association of physical activity and sedentary time with cfPWV and VAI, several models of multiple linear regression analysis was performed. cfPWV (m/s) and VAI were considered as dependent variables and total physical activity, sedentary time (min/week), number of steps per day, sedentary time measured in hours/week, were considered as independent variables. Age (years), sex (women = 0 and men = 1), years of smoking, alcohol consumption in g/week, Mediterranean diet adherence score, diastolic blood pressure in mmHg, body mass index in (kg/$m^{2}$), lipid-lowering drugs (No = 0 and Yes = 1) and hypoglycaemics drugs (No = 0 and Yes = 1) were the adjustment variables. To explore the association of physical activity and sedentary time with the degree of vascular aging, estimated with the cfPWV and VAI percentiles, we performed several multinomial logistic regression models (coded as HVA = 1, NVA = 2, and EVA = 3), using HVA as the reference value. Total physical activity, sedentary time (min/week), number of steps per day, sedentary time measured in hours/week, and intensity of physical activity of subjects were classified (light physical activity = 1, moderate physical activity = 2 and vigorous physical activity = 3), using light physical activity as the reference value, were considered as independent variables. Using adjustment variables the same as in multiple regression models. All the analyses were conducted with the complete sample and by sex. In the hypothesis test, statistical significance was set at *p* = 0.05. The statistical software used in the analysis was SPSS software for Windows, v25.0 (IBM Corp, Armonk, NY, USA).

## 3. Results

### 3.1 Characteristics of the Participants

The general characteristics of the analyzed individuals, globally and by sex, are presented in Table 1, including lifestyles, cardiovascular risk factors, parameters of vascular structure and function, physical activity, and sedentary time. The average age was 55.90 years with a SD of 14.24 years. Men consumed more alcohol and showed worse adherence to the Mediterranean diet. The mean values of cIMT and cfPWV showed higher values in men. The time spent performing total physical activity and light physical activity recorded with the accelerometer for one week was longer in women, whereas moderate–vigorous physical activity, number of steps per day, and sedentary time showed greater values in men.

**Table 1.** **General characteristics of the subjects**.

	Global (n = 501)	Men (n = 249)	Women (n = 252)	*p* value
Lifestyles				
Alcohol (g/w)	80.8 ± 68.7	98.2 ± 72.7	52.1 ± 49.6	<0.001
Excessive alcohol consumption, n (%)	50 (10.0)	34 (13.70)	16 (6.30)	0.006
Smoker, n (%)	90 (18.0)	49 (19.7)	41.0 (16.3)	0.190
Smoker (years)	29.2 ± 14.4	31.5 ± 15.5	26.7 ± 12.8	0.012
Mediterranean diet score	7.2 ± 2.1	6.7 ± 1.9	7.7 ± 6.1	<0.001
Adherence to the MD, n (%)	127 (25.3)	42 (16.9)	83 (33.7)	<0.001
Conventional risk factors				
Age (years)	55.9 ± 14.2	55.9 ± 14.3	55.8 ± 14.2	0.935
Systolic blood pressure (mmHg)	122 ± 23	126 ± 20	115 ± 25	<0.001
Diastolic blood pressure (mmHg)	76 ± 10	77 ± 9	74 ± 10	<0.001
Hypertensive, n (%)	147 (25.8)	82 (32.9)	65 (29.3)	<0.001
Antihypertensive drugs, n (%)	96 (19.2)	50 (20.1)	46 (18.3)	0.650
Total cholesterol (mg/DL)	195 ± 32	193 ± 32	197 ± 33	0.142
LDL cholesterol (mg/DL)	115 ± 29	117 ± 14	114 ± 28	0.148
HDL cholesterol (mg/DL)	59 ± 16	53 ± 14	64 ± 28	<0.001
Triglycerides (mg/DL)	103 ± 53	112 ± 54	94 ± 50	<0.001
Dyslipidaemia, n (%)	191 (38.1)	95 (38.1)	96 (38.2)	0.989
Lipid-lowering drugs, n (%)	102 (20.4)	49 (19.7)	53 (21.0)	0.396
Fasting plasma glucose (mg/DL)	88 ± 17	90 ± 19	86 ± 16	0.013
HbA1c, (%)	5.49 ± 0.56	5.54 ± 0.63	5.44 ± 0.47	0.044
Diabetes mellitus, n (%)	38 (7.6)	26 (10.5)	12 (4.8)	0.012
hypoglycaemics drugs, n (%)	35 (7.0)	23 (9.2)	12 (4.8)	0.055
Body mass index (kg/$m^{2}$)	26.52 ± 4.23	26.90 ± 4.08	26.14 ± 4.79	0.044
Obesity, n (%)	94 (18.8)	42 (16.9)	52 (20.6)	0.304
Vascular structure and function				
cIMT (mm)	0.680 ± 0.109	0.696 ± 0.116	0.665 ± 0.100	0.002
cfPWV (m/s)	8.15 ± 2.49	8.54 ± 2.68	7.71 ± 2.23	0.001
VAI	61.04 ± 12.77	63.12 ± 13.66	59.04 ± 11.54	<0.001
Physical activity and sedentary time				
Total PA (m/W)	1625 ± 571	1560 ± 570	1690 ± 565	0.011
Low intensity PA (m/W)	1237 ± 482	1136 ± 476	1339 ± 468	<0.001
Moderate and High intensity PA (m/W)	387 ± 226	423 ± 246	351 ± 199	<0.001
Steps per day (number)	9295 ± 4194	9861 ± 4482	8727 ± 3810	0.003
Sedentary time (hours/W)	141 ± 10	142 ± 10	140 ± 9	0.017

Excessive alcohol consumption in women was ≥140 g/week and ≥210 g/week in men. The *p* value indicates differences between men and women. g/w, grams/week; MD, Mediterranean diet; LDL, low-density lipoprotein; HDL, high-density lipoprotein; HbA1c, glycosylated haemoglobin; cIMT, carotid intima-media thickness; cfPWV, carotid-to-femoral aortic pulse wave velocity; VAI, vascular aging index; PA, physical activity; m/W, minutes/week. *p* value: differences between men and women.

### 3.2 Association of Vascular Aging with Physical Activity and Sedentary Time Globally and by Sex

The mean values of time spent performing physical activity and sedentary time in the individuals classified as HVA, NVA, and early vascular aging globally and by sex using cfPWV are shown in Table 2, and those obtained using VAI are shown in Table 3. The results of the two criteria were similar, although steps per day only reached a statistically significant difference when using cfPWW (*p* = 0.048). The analyses by sex showed the same tendencies, although statistical significance was only reached in men when cfPWV was used.

**Table 2.** **Characteristics of physical activity and sedentary time in participants with healthy, normal, and early vascular aging, globally and by sex, using the 25th and 75th percentiles of cfPWV**.

		HVA	NVA	EVA	*p* value
Global		(19.1%)	(48.6%)	(32.3%)	
	Total PA (m/W)	1722 ± 566	1648 ± 563	1531 ± 579	0.025
	Steps per day (number)	10,102 ± 4035	9310 ± 4133	8759 ± 4350	0.048
	Sedentary time (hours/W)	140 ± 9	142 ± 10	141 ± 10	0.017
Men		(16.2%)	(49.0%)	(34.8%)	
	Total PA (m/W)	1700 ± 593	1602 ± 559	1440 ± 551	0.032
	Steps per day (number)	11,116 ± 3974	9987 ± 4456	9058 ± 4470	0.050
	Sedentary time (hours/W)	139 ± 10	141 ± 10	144 ± 9	0.018
Women		(21.9%)	(48.2%)	(29.9%)	
	Total PA (m/W)	1739 ± 551	1694 ± 555	1639 ± 596	0.612
	Steps per day (number)	9350 ± 3950	8628 ± 3673	8408 ± 3944	0.364
	Sedentary time (hours/W)	140 ± 9	140 ± 9	141 ± 10	0.619

The differences among the groups were analyzed by ANOVA. The *p* value indicates the differences between the groups according to whether they are classified as HVA, NVA, or EVA. HVA, healthy vascular aging; NVA, normal vascular aging; EVA, early vascular aging; cfPWV, carotid-to-femoral aortic pulse wave velocity; PA, physical activity; ANOVA, analyses of variance; m/W, minutes/week.

**Table 3.** **Characteristics of physical activity and sedentary time in participants with healthy, normal, and early vascular aging, globally and by sex, using the 25th and 75th percentiles of VAI**.

		HVA	NVA	EVA	*p* value
Global		(18.9%)	(48.8%)	(32.3%)	
	Total PA (m/W)	1752 ± 588	1617 ± 558	1561 ± 574	0.037
	Steps per day (number)	10,228 ± 4167	9107 ± 4075	8998 ± 4363	0.053
	Sedentary time (hours /W)	138 ± 9	141 ± 10	141 ± 10	0.027
Men		(17.8%)	(46.6%)	(35.6%)	
	Total PA (m/W)	1683 ± 605	1573 ± 565	1486 ± 560	0.170
	Steps per day (number)	10,832 ± 4342	9724 ± 4417	9511 ± 4653	0.261
	Sedentary time (hours /W)	141 ± 10	143 ± 9	142 ± 10	0.114
Women		(19.9%)	(51.0%)	(29.1%)	
	Total PA (m/W)	1814 ± 571	1657 ± 551	1654 ± 581	0.219
	Steps per day (number)	9685 ± 3970	8548 ± 3667	8363 ± 3915	0.135
	Sedentary time (hours /W)	140 ± 10	141 ± 10	143 ± 9	0.226

The differences among the groups were analyzed by ANOVA. The *p* value indicates differences between the groups according to whether they are classified as HVA, NVA, or EVA. HVA, healthy vascular aging; NVA, normal vascular aging; EVA, early vascular aging; VAI, vascular aging index; PA, physical activity; ANOVA, analyses of variance; m/W, minutes/week.

### 3.3 Association of Physical Activity and Sedentary Time with Arterial Stiffness

Table 4 shows the multiple regression analysis globally and by sex. We found a negative association of total physical activity showed with cfPWV (β = –0.454; 95% confidence interval [CI] –0.733 to –0.174) and with VAI (β = –1.845; 95% CI –3.091 to –0.599). The number of steps per day showed a negative association with cfPWV (β = –0.052; 95% CI –0.092 to –0.013) and with VAI (β = –0.216; 95% CI –0.389 to –0.044); and sedentary time showed a positive association with cfPWV (β = 0.028; 95% CI 0.002 to 0.045) and with VAI (β = 0.117; 95% CI 0.042 to 0.191). In the analysis by sex, the results were similar. **Supplementary Table 2** shows the same results using the global analysis, only age and sex as the adjustment variables, and only age in the analysis by sex.

**Table 4.** **Multiple regression analysis, globally and by sex, of arterial stiffness with physical activity and sedentary time**.

		β cfPWV	95% CI		*p* value	β VAI	95% CI		*p* value
Global									
	Total PA (m/W)	–0.454	–0.733	–0.174	0.002	–1.845	–3.091	–0.599	0.004
	Steps day (number)	–0.052	–0.092	–0.013	0.009	–0.216	–0.389	–0.044	0.014
	Sedentary time (hours/W)	0.028	0.002	0.045	<0.001	0.117	0.042	0.191	0.002
Men									
	Total PA (m/W)	–0.490	–0.911	–0.069	0.023	–1.613	–3.427	0.159	0.031
	Steps day (number)	–0.054	–0.109	0.002	0.058	–0.173	–0.412	0.066	0.155
	Sedentary time (hours/W)	0.032	0.006	0.057	0.014	0.106	0.001	0.214	0.048
Women									
	Total PA (m/W)	–0.423	–0.787	–0.058	0.023	–2.088	–3.772	–0.400	0.016
	Steps day (number)	–0.070	–0.127	–0.013	0.016	–0.371	–0.629	–0.113	0.005
	Sedentary time (hours/W)	0.026	0.004	0.646	0.022	0.127	0.026	0.228	0.014

Multiple regression analysis using cfPWV m/s and VAI as dependent variables. Steps per day, total physical activity (m/W), and sedentary time in hours per week as independent variables, and age (years), sex (women = 0 and men = 1), years of smoking, alcohol consumption in g/week, mediterranean diet adherence score, diastolic blood pressure in mmHg, body mass index in (kg/$m^{2}$), lipid-lowering drugs (No = 0 and Yes = 1) and hypoglycemics drugs (No = 0 and Yes = 1) as adjustment variables. β, standardized regression coefficient; cfPWV, carotid-to-femoral aortic pulse wave velocity; VAI, vascular aging index; PA, physical activity; m/W, minutes per week; 95% CI, 95% confidence interval. The *p*-value indicates whether there is an association between cfPWV or VAI with steps per day, physical activity, and sedentary time in hours per week after adjusting for possible confounding variables.

### 3.4 Association of Physical Activity and Sedentary Time with Vascular Aging

**Fig. 2.**
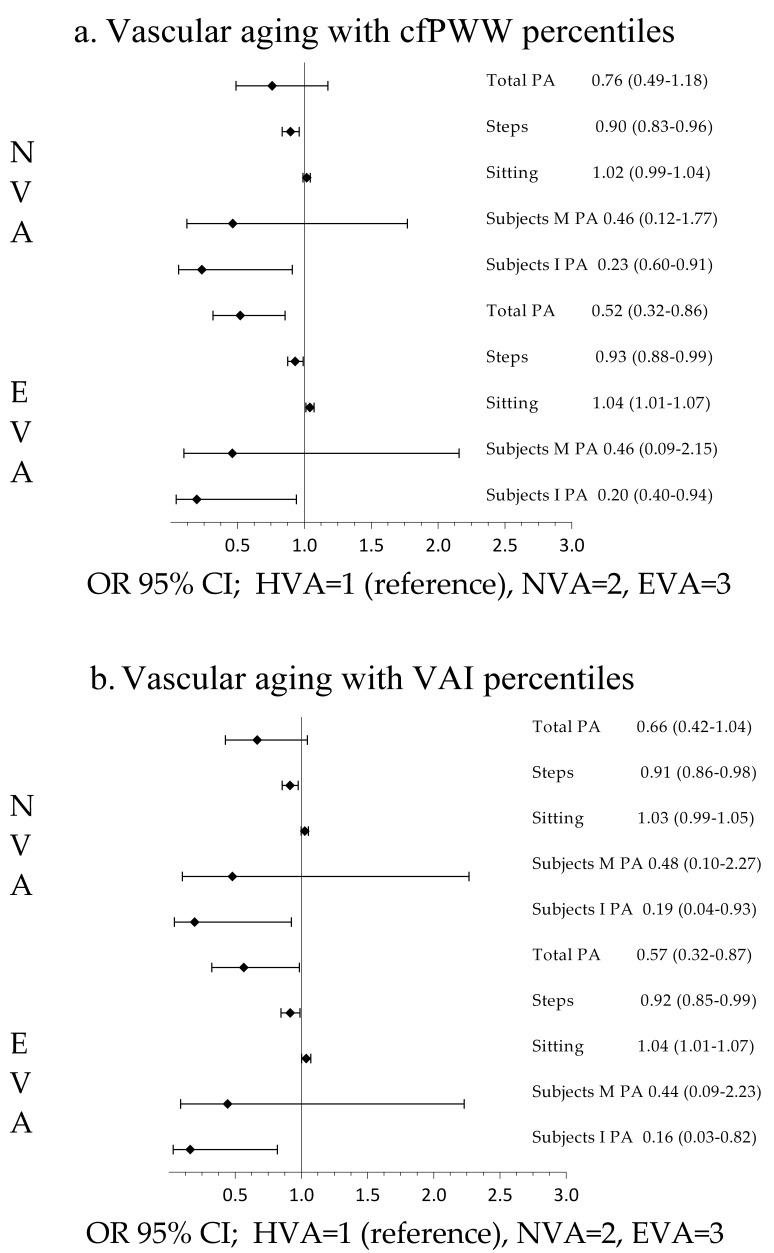
**Global association of sedentary time and physical activity with vascular aging**. Multinomial logistic regression analysis using (a) cfPWV and (b) VAI. VAI, vascular aging index; cfPWV, carotid-to-femoral aortic pulse wave velocity; PA, physical activity; M PA, moderate physical activity; I PA, intense physical activity; HVA, healthy vascular aging; NVA, normal vascular aging; EVA, early vascular aging; OR, odds ratio; CI, confidence interval.

**Fig. 3.**
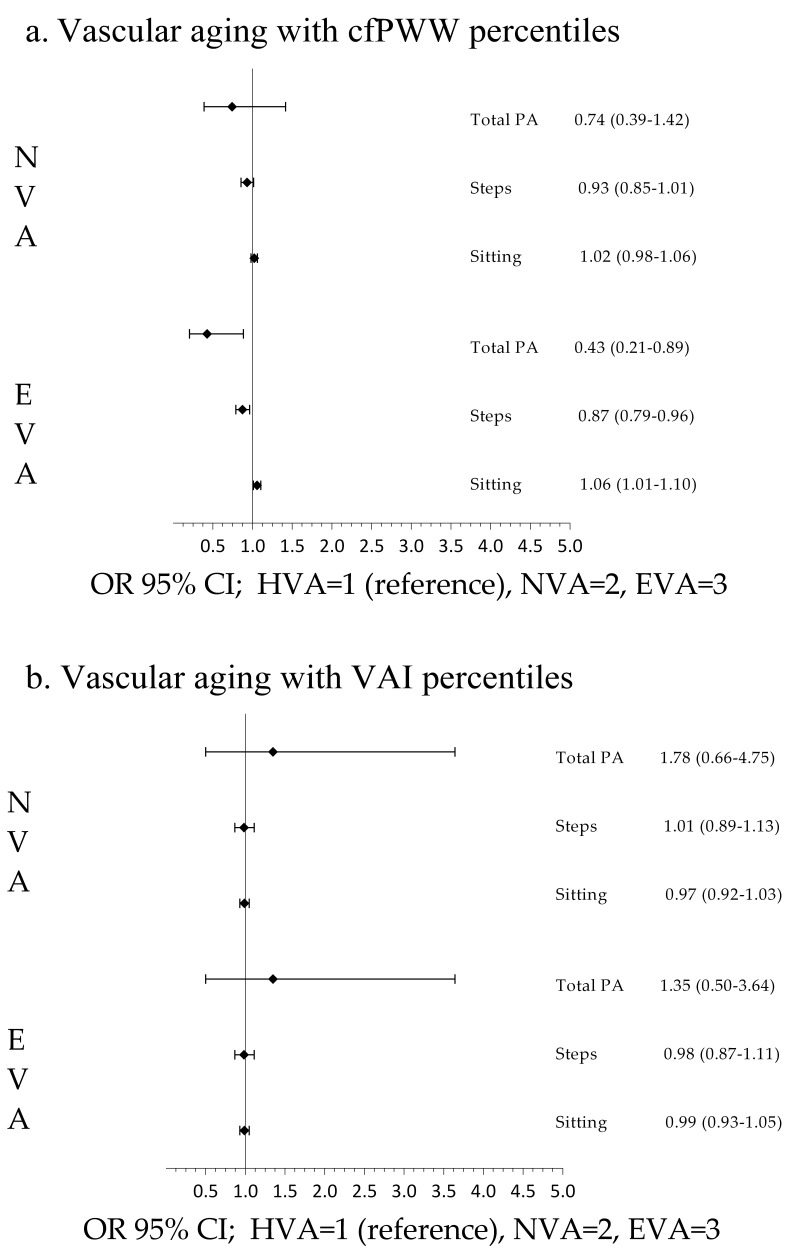
**Association of sedentary time and physical activity with vascular aging in men**. Multinomial logistic regression analysis using (a) cfPWV and (b) VAI. VAI, vascular aging index; cfPWV, carotid-to-femoral aortic pulse wave velocity; PA, physical activity; HVA, healthy vascular aging; NVA, normal vascular aging; EVA, early vascular aging; OR, odds ratio; CI, confidence interval.

**Fig. 4.**
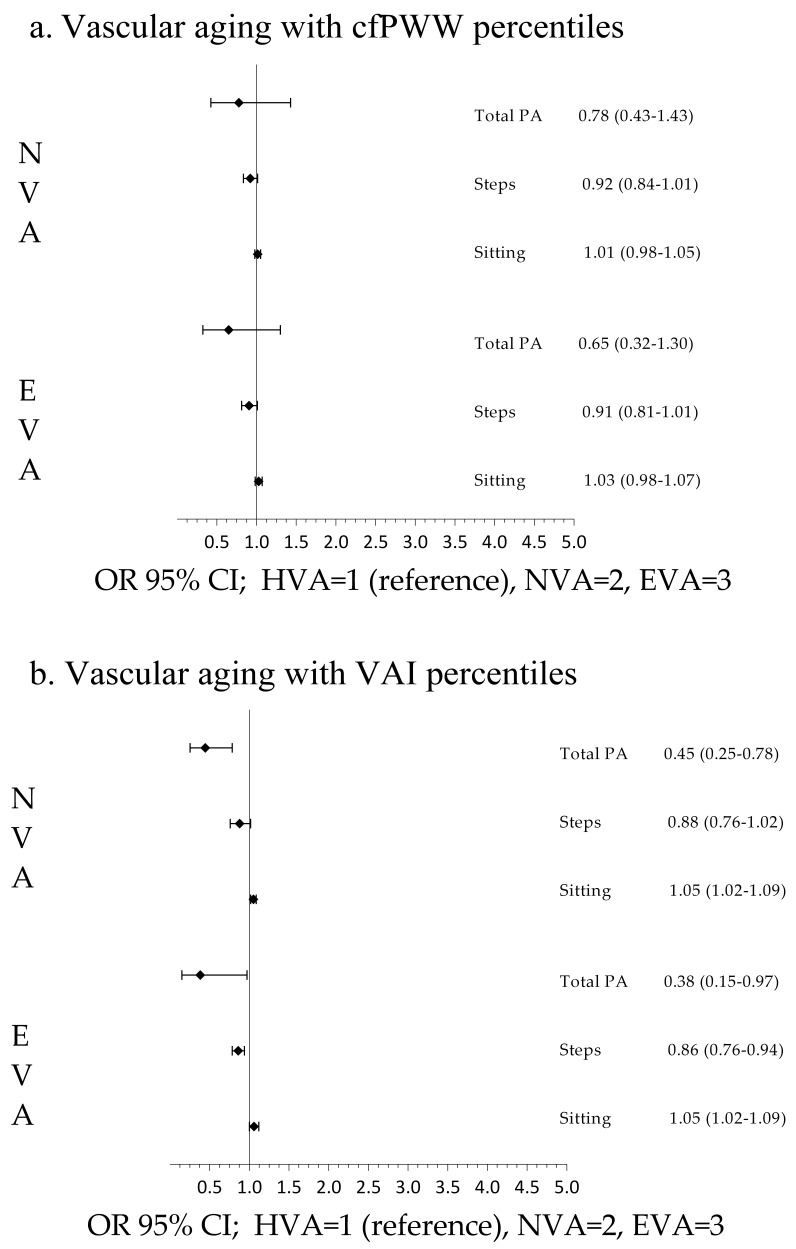
**Association of sedentary time and physical activity with vascular aging in women**. Multinomial logistic regression analysis using (a) cfPWV and (b) VAI. VAI, vascular aging index; cfPWV, carotid-to-femoral aortic pulse wave velocity; PA, physical activity; HVA, healthy vascular aging; NVA, normal vascular aging; EVA, early vascular aging; OR, odds ratio; CI, confidence interval.

The results of the multinomial logistic regression analysis, after controlling for possible confounding factors, are shown globally (Fig. 2), in men (Fig. 3), and in women (Fig. 4). For the entire sample, the odds ratio (OR) of total physical activity of the individuals classified as early vascular aging, with respect to those classified as HVA and using cfPWV to define the degree of vascular aging, was 0.521 (95% CI 0.317 to 0.856), and 0.565 (95% CI 0.324 to 0.986) using VAI. The OR of the number of steps per day was 0.931 (95% CI 0.875 to 0.992) using cfPWV and 0.916 (95% CI 0.847 to 0.990) using VAI. The OR of sedentary time was 1.042 (95% CI 1.011 to 1.073) using cfPWV and 1.037 (95% CI 1.003 to 1.072) using VAI. The OR of subjects classified as vigorous physical activity was 0.196 (95% CI 0.041 to 0.941) using cfPWV and 0.194 (95% CI 0.041 to 0.916) using VAI. The OR of vigorous physical activity of the individuals classified as NVA, with respect to those classified as HVA and using cfPWV was 0.070 (95% CI 0.020 to 0.280) using VAI was 0.161 (95% CI 0.032 to 0.820). In the analysis by sex, the results showed an association in men when cfPWV was used (Fig. 3) and an association in women when VAI was used to define vascular aging.

## 4. Discussion

The main findings determined a negative association between the total number of minutes of physical activity per week and the number of steps per day with arterial stiffness measured objectively with an accelerometer. The number of hours spent sitting per week showed a positive association with arterial stiffness. In the multinomial regression analysis, subjects classified as EVA with respect to those classified as HVA showed associations in the same sense and subjects classified as NVA with respect to those classified as HVA showed associations in vigorous physical activity in subjects without previous cardiovascular disease. In the analysis by sex, the association is maintained in men when cfPWV was used and in women when VAI was used to define vascular aging.

The results of this study highlight that the time spent performing total physical activity and light physical activity recorded with the accelerometer for one week was greater in women, whereas moderate or vigorous physical activity, the number of steps per day, and sedentary time showed higher values in men. These results are in line with the results published in European populations by Giné-Garriga *et al*. [49], who also found time spent performing total physical activity and light physical activity recorded with the accelerometer for one week to be greater in women, whereas moderate or vigorous physical activity, the number of steps per day, and sedentary time again showed greater values in men; and by Mattle *et al*. [50] who found that males had greater compliance with physical activity recommendations defined as performing ≥150 min/week of moderate physical activity and/or ≥75 min/week of vigorous physical activity.

Previous studies have found similar results indicating that physical activity improves arterial stiffness. Moreover, the beneficial effect of physical exercise on different aspects of health has been demonstrated in several studies, in different groups of patients measuring physical activity both objectively and subjectively [14, 51, 52]. Nevertheless, some studies only found benefits on arterial stiffness when arterial stiffness was high intensity [19, 20]. Vandercappellen *et al*. [18] have recently found an association between high intensity physical activity and lower arterial stiffness, with no differences between exercise patterns. From an arterial stiffness perspective, this could indicate that doing intense physical activity, irrespective of any weekly schedule, might represent a significant strategy in reducing cardiovascular disease risks. Several prospective studies, both in the general population and in people with cardiovascular risk factors, have found that increased arterial stiffness increases morbidity and mortality from cardiovascular disease, as well as total mortality. The results of these studies have been reported in two meta-analyses [53, 54]. Vlachopoulos *et al*. [54] concluded that for every meter per second increase in cfPWW, cardiovascular risk increased by 14% for total cardiovascular events, and by 15% for cardiovascular morbidity and all-cause mortality. Ben-Shlomo *et al*. [53] concluded that, for each one SD increase in log cfPWW, the OR for coronary heart disease was 1.23 (95% CI 1.11–1.35), 1.28 (95% CI 1.16–1.42) for stroke, and 1.30 (95% CI 1.18–1.43) for cardiovascular events. Similarly, aerobic and resistance exercise was shown to diminish arterial stiffness in a clinical trial meta-analysis of 2089 patients [35]. Therefore, the available evidence suggests that the more time we spend on physical activity, especially if it is of moderate or vigorous intensity, the lower the stiffness of the arteries and therefore the healthier vascular aging will be [13]. The same was observed for the number of steps per day, whose increase reduced arterial stiffness. Cabral *et al*. [16] have recently reported that every increase of 1000 steps/day is associated with a decrease of 0.05 m/s in cfPWV. In addition, the greater the cadence, the lower the cfPWV [55].

Other researchers have already discussed the correlation between vascular function metrics and periods of inactivity. They examined cardiovascular function in sedentary adults engaged in various physical exercises [56, 57, 58]. Ahmadi *et al*. [59] have shown an association between reducing sedentary periods and slowing the progression of age-related stiffness in the aorta. This outcome corroborates the findings of the present research, implying that those with greater sedentary time are more likely to fall into the early vascular aging category. In this context, multiple studies have indicated a link between time spent watching television and worse cardiovascular well-being [60, 61]. Wennman *et al*. [62] calculated that the likelihood of developing cardiovascular disease, as determined by Framingham’s formula, was greater among men watching TV for four hours or more every day, and among women with two to three hours of daily TV consumption. The harmful effect of sedentary behaviors on cardiometabolic and obesity-related traits does not depend on the levels of physical activity. Therefore, reducing sedentary time must be a goal of the population, as well as increasing their levels of physical activity [1]. Nevertheless, sedentary lifestyles are expanding worldwide, probably due to the lack of spaces available for exercising, the increase of occupational sedentary behaviors (e.g., office work), and the use of leisure time to watch TV and browse the Internet [8].

In agreement with the findings of this research, several studies have shown how vascular aging is linked to increased physical activity along with reduced sedentary behavior [23, 38, 63]. Increasing physical activity could therefore counteract the adverse impacts of aging on vascular function [13]. Moreover, long sedentary time is associated with other cardiovascular risk factors [64, 65], irrespective of physical activity levels. A study in Australia, for example, observed that with each added hour of sedentary time the correlation with cardiovascular risk factors in men and women rose by 5% and 4%, respectively [66]. Despite these findings, studies conducted in Europe, the United States, and Australia have shown that adults are seated for 50% of the workday (4.2 h/day) and for about 2.9 hours per day in their leisure time [67].

Nevertheless, not all studies have found the same results, and there are discrepancies between studies that analyze the effect of physical activity, its intensity, and sedentary time on arterial stiffness and aging in global analyses [18, 19, 20] and analyses by sex [21, 22]. The discrepancies between the different studies could be due, at least partially, to the inclusion of individuals of different ages, the subjective measuring of physical activity through questionnaires, the objective measuring of physical activity using an accelerometer, and the measuring of arterial stiffness with different devices, as well as the use of different criteria to define the degree of vascular aging.

Hypertension and arterial stiffness are closely related, and the concurrence of the two is complex [67, 68]. Moreover, the technique of measuring blood pressure by oscillometry is influenced by age [69]. Thus, devices that allow the measurement of these parameters in real time should be taken into account in future research.

The main limitation of this work was, firstly, the cross-sectional nature of the analysis, which does not allow an inference of causality. Secondly, the results of this study refer only to the Spanish population in the age range of 35–75 years without previous cardiovascular disease; therefore, our results cannot be generalized to other groups or races/ethnicities. In addition, it is noteworthy that pulse waveform can be influenced by filtering in preprocessing, which depends on the measurement site and the physiological conditions of the patient [70]. Regarding the main strengths of this study, the sample was obtained through simple random sampling, and physical activity and sedentary time were measured objectively using an accelerometer for one week. The main contributions of this work are that, as far as we know, it is the first study carried out in a sample of the Spanish adult population without previous cardiovascular disease, which analyses the relationship of the time spent performing physical activity, its intensity, and sitting time, measured objectively by accelerometry, with vascular aging. Two criteria were used in the definition of vascular aging: one little-known method of combining the parameters of vascular structure and function using the cfPWV and cIMT percentiles, and another more frequently used method based on the criteria of vascular function using the cfPWV percentiles.

## 5. Conclusions

The results of this study suggest that the greater the time spent doing vigorous physical activity the lower the probability of developing early vascular aging as assessed by cfPWV and by VAI. Likewise, the longer the sedentary time, the higher the probability of developing early vascular aging, assessed by cfPWV and by VAI. Therefore, a beneficial strategy for achieving healthy aging in adults would be more movement and less sitting.

## Data Availability

The datasets used and/or analysed during the present study are available upon reasonable request to the corresponding author.

## Supplementary Material

Supplementary material associated with this article can be found, in the online version, at https://doi.org/10.31083/j.rcm2411318.
