# Supplementary material for: Association of Accelerometer-Measured Sedentary Time and Physical Activity with Arterial Stiffness and Vascular Aging in the General Spanish Population, Analyzed by Sex
Source: Rev Cardiovasc Med. 2023 Nov 17;24(11):318. doi: 10.31083/j.rcm2411318 (PMC11272887; doi:10.31083/j.rcm2411318)
Supplement: Supplementary file 1 [file 2153-8174-24-11-318-s1.pdf]

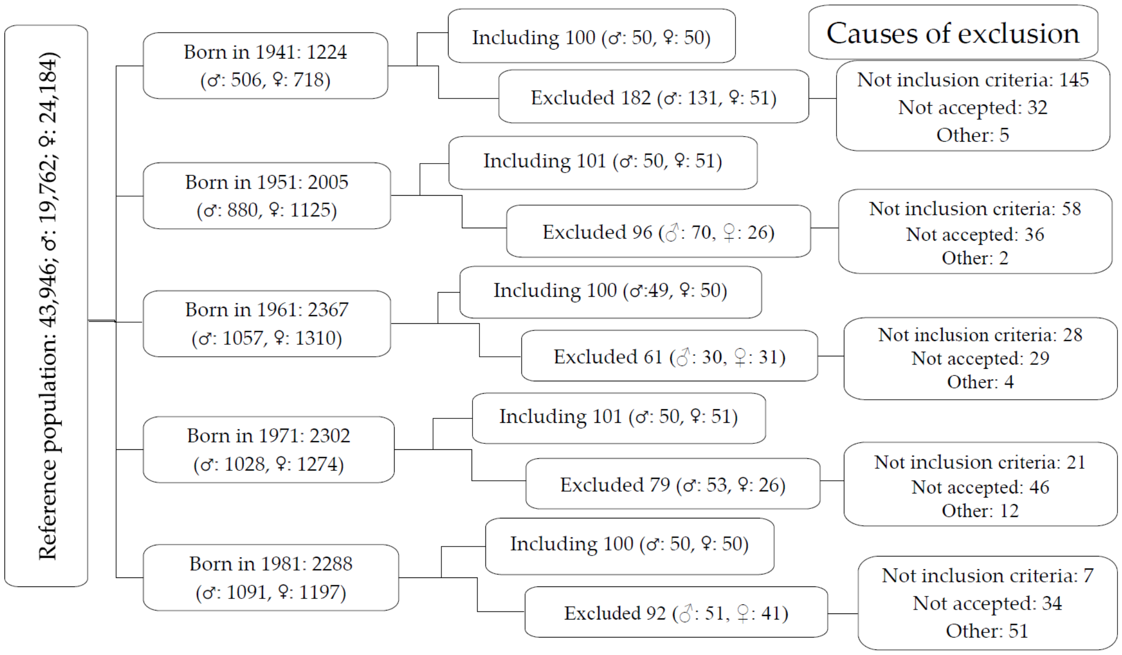

Additional file 1: Supplementary Fig. 1. Flowchart of the study. It indicates the reference population by age and sex, the individuals included and excluded, and the main causes of exclusion. A total of 259 individuals did not meet the inclusion criteria. A total of 177 individuals did not wish to participate in the study, and 74 individuals could not be contacted for changing either their address or phone number. The replacement rate
was 35.4% and the response rate was 64.6%. ♂: men; ♀: women.

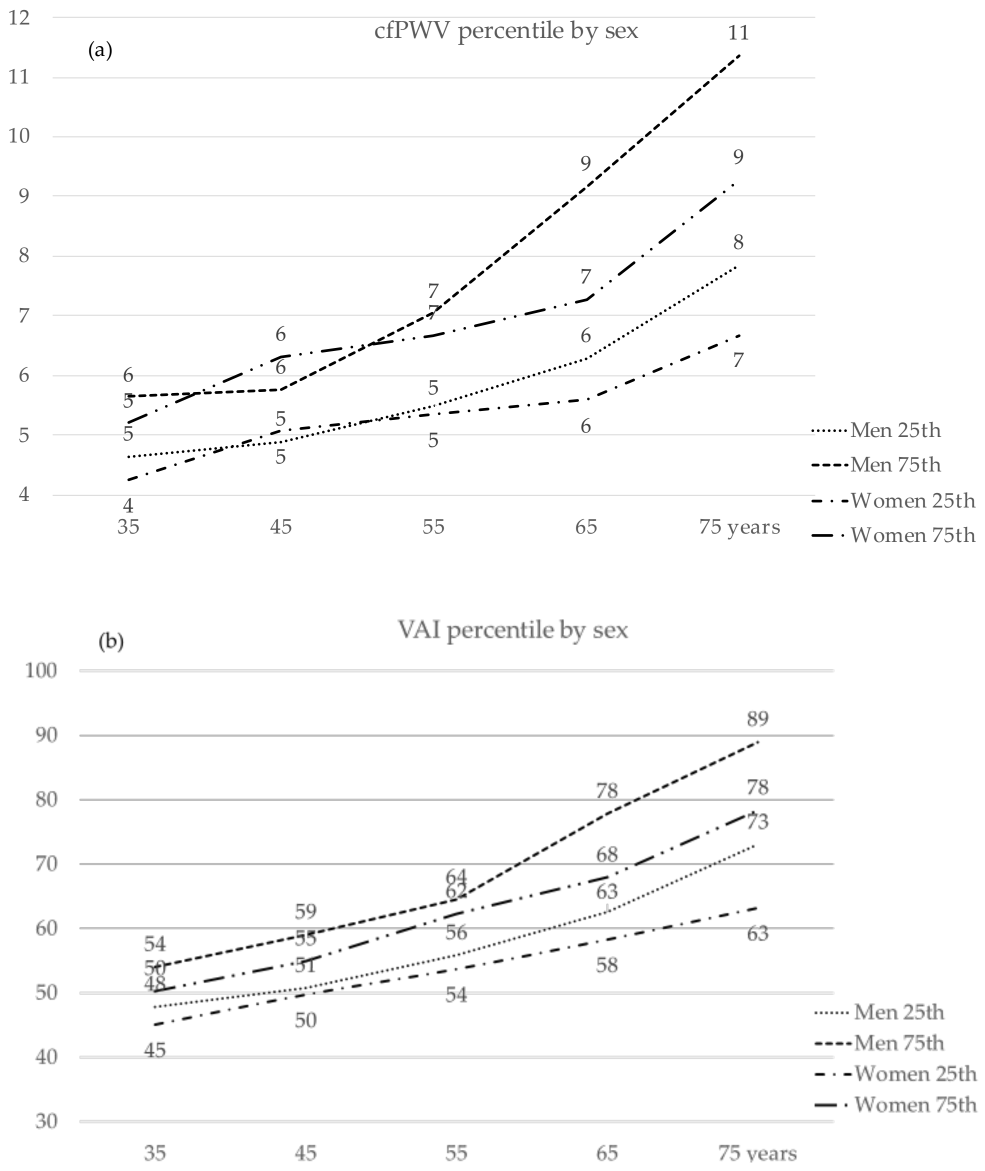

Additional file 2: Supplementary Fig. 2. Age-specific 25th, 75th, (a) percentile for carotid-femoral pulse wave velocity and by (b) percentile for vascular ageing index in participants without prevalent cardiovascular disease. cfPWV, carotid-femoral aortic pulse wave velocity; VAI, vascular ageing index; P, percentile.

Additional File 3: Supplementary Table 1. The questionnaire of Adherence to the Mediterranean diet

Nº	Question	Assessment
1	Do you use olive oil as the principal source of fat for cooking?	Yes =1 point
2	Do you consume ≥ 4 tablespoon of olive oil per day?	Two or more tablespoons = 1 point
3	Do you consume ≥ 2 servings of vegetables per day?	Two or more a day (at least one of them in salads or raw)= 1 point
4	Do you consume ≥3 pieces of fruit per day?	Three or more a day = 1 point
5	Do you consume < 1 serving of red meat, hamburger, or sausages per day?	Less than one a day = 1 point
6	Do you consume < 1 serving of butter, margarine, or cream per day?	Less than one a day = 1 point
7	Do you consume < 1 carbonated and/or sugar-sweetened beverages per day?	Less than one a day = 1 point

8	Do you drink ≥ 7 cups (100 ml) of wine per week?	Three or more glasses per week = 1 point
9	Do you consume ≥ 3 servings of pulses per week?	Three or more per week = 1 point
10	Do you consume ≥ 3 servings of fish/seafood per week?	Three or more per week = 1 point
11	Do you consume < 2 commercial pastry such as cookies or cake per week?	Less than three per week = 1 point
12	Do you consume ≥ 3 servings of nuts per week?	One or more per week = 1 point
13	Do you prefer to eat chicken, turkey, or rabbit instead of beef, pork, hamburgers, or sausages?	Yes =1 point
14	Do you consume ≥ 2 times per week boiled vegetables, pasta, rice, or other dishes with a sauce of tomato, garlic, onion, or leeks sautéed in olive oil?	Two or more per week = 1 point

TOTAL SCORE: < 9 low adherence ≥ 9 good adhesion

Additional file 4: Supplementary Table 2. Multiple regression analysis, in global and by sex, of arterial stiffness with physical activity and sedentary time.

Supplementary Table 2 Multiple regression analysis in global and by sex of arterial stiffness with physical activity and sedentary time.

	**β**	**95%**	**CI**	**p value**	**β**	**95%**	**CI**	**p value**
**Global**	**cfPWV**				**VAI**			
Total PA, (m/W)	-0.378	-0.615	-0.141	0.002	-2.023	-3.356	-0.689	0.003
Steps day, (number)	-0.043	-0.076	-0.011	0.009	-0.248	-0.429	-0.066	0.008
Sedentary time, (m/W)	0.397	0.162	0.633	0.001	0.120	0.035	0.206	0.006
**Men**								
Total PA, (m/W)	-0.426	-0.783	-0.070	0.019	-2.023	-3.356	-0.689	0.003
Steps day, (number)	-0.051	-0.098	-0.005	0.031	-0.248	-0.429	-0.066	0.008
Sedentary time, (m/W)	0.462	0.108	0.815	0.011	0.120	0.035	0.206	0.006
**Women**								
Total PA, (m/W)	-0.341	-0.651	-0.030	0.032	-1.847	-3.799	0.105	0.063
Steps day, (number)	-0.048	-0.094	-0.002	0.041	-0.236	-0.490	0.019	0.070
Sedentary time, (m/W)	0.339	0.029	0.649	0.032	0.177	0.046	0.309	0.008

Multiple regression analysis using cfPWV m/sec and VAI as dependent variables. Steps day, Physical activity and sedentary time minutes per week as independent variables, and age (years), sex (women = 0 and men = 1), as adjustment variables.
β, Standardized regression coefficient; cfPWV, carotid-to-femoral aortic pulse wave velocity; VAI, vascular aging index; PA, physical activity; m/W, minute/week.
